# Influence of the Noise-Canceling Technology on How We Hear Sounds

**DOI:** 10.3390/healthcare10081449

**Published:** 2022-08-02

**Authors:** Hye-Yoon Seol, Seo-Hu Kim, Ga-Young Kim, Mini Jo, Young-Sang Cho, Sung-Hwa Hong, Il-Joon Moon

**Affiliations:** 1 Medical Research Institute, Sungkyunkwan University School of Medicine, Suwon 16419, Korea; seol.helena@gmail.com; 2 Hearing Research Laboratory, Samsung Medical Center, Seoul 06351, Korea; clementine21@naver.com (S.-H.K.); kgyslp@gmail.com (G.-Y.K.); minij1231@gmail.com (M.J.); divetosun@gmail.com (Y.-S.C.); hongsh@mjh.or.kr (S.-H.H.); 3 Samsung Advanced Institute for Health Sciences & Technology, Sungkyunkwan University, Seoul 06355, Korea; 4 Department of Otolaryngology-Head & Neck Surgery, Samsung Medical Center, Sungkyunkwan University School of Medicine, Seoul 06351, Korea; 5 Department of Otolaryngology-Head & Neck Surgery, Samsung Changwon Hospital, Sungkyunkwan University School of Medicine, Changwon 51353, Korea

**Keywords:** hearing loss, noise, noise-induced hearing loss

## Abstract

Purpose: This study explores the influence of the noise-canceling technology in commercial earphones on sound pressure levels and preferred listening levels in terms of hearing protection. Materials and Methods: Thirty individuals completed puretone audiometry and real-ear measurements to assess sound pressure levels at the level of the eardrum with and without the activation of the noise-canceling function. The Knowles Electronics Manikin for Acoustic Research was used to investigate the acoustic characteristics of two environmental sounds (bus and café). Two types of earphones (wireless and wired canal type) were utilized in the study. Results: For both bus and café noises, in the low-frequency range, sound pressure levels were significantly decreased for all types of earphones when the noise-canceling function was turned on. The same results were observed for the whole frequency range. In terms of preferred listening levels, the wireless canal type and wired canal type earphones showed significant reduction in volume levels. Conclusion: The findings of the study show that for both low- and whole frequency range, the use of noise-canceling function significantly decreases the sound pressure levels of the signal for all styles of earphones, suggesting a potential of the noise-canceling technology in hearing protection.

## 1. Introduction

Individuals are exposed to various types of noise in life. Types of noise range from traffic noise to orchestral noise [1,2]. The loudness of these noises is typically louder than conversations, which is approximately 60 dBA. For instance, the average levels of subway and street noise were reported to be 86 and 70–80 dBA, respectively [3,4]. With the increased prevalence of personal listening devices, the health risks for individuals, especially young adults and teenagers, have increased as well. Exposure to loud sounds is related to safety issues [2]. For example, walking while listening to music could lead to crashes because people are not able to pay attention to their surroundings [5]. Schwebel et al. (2012) conducted an observational study of 138 students. The students were randomly divided into four groups (phone, texting, personal music device, and undistracted), and they were asked to cross a virtual street. Compared to the control group (undistracted), those who were in the texting and personal music-listening device groups experienced more hits while crossing the street [5]. In terms of hearing, prolonged noise exposure has been associated with the onset of noise-induced hearing loss [6,7]. The World Health Organization reported in 2019 that about 40% of teenagers and young adults are listening to sound at a level that is potentially damaging their hearing [7]. Hussain et al. (2018) examined young adults’ hearing acuity as well as preferred volume levels in various noise conditions [8]. Fifty individuals who used an iPod or iPhone on a regular basis were enrolled in the study and completed a questionnaire assessing average hours of use and preferred volume levels. As an objective measure, the participants’ preferred volume output levels were also examined in different noise conditions in a sound booth. Among the 50 participants, 11 reported dangerous listening behaviors, which included a preferred volume output level of 91 dBA or greater as well as an average of two hours or more for device use. Regarding hearing thresholds, elevated thresholds were observed at 4000 and 6000 Hz for these individuals. In terms of preferred listening levels (PLL), those higher than the conversational level (60 dBA) were reported by several pieces of the literature [4,9]. For example, the mean PLL was reported to be 86.1 dBA for the street noise of 73.2 dBA in Williams (2005) [4]. Another study explored the PLLs of 38 individuals with normal hearing based on earphone style (earbud and over-the-ear) and listening conditions [9]. Each individual’s PLL was assessed in quiet, street noise (70–80 dBA), and multi-talker babble (70 dBA) conditions. Once the PLLs were obtained, real-ear measurements (REMs) were performed to see the output levels of the PLLs. While the mean sound pressure levels ranged from 75.2 to 77.8 dBA for the earbud and over-the-ear earphone styles in quiet, the mean sound pressure levels ranged from 83.0 to 88.8 dBA in the street noise condition for both styles. For the multi-talker babble noise, the mean sound pressure levels for the earphone styles ranged from 81.6 to 86.7 dBA.

In South Korea, Byeon (2021) explored the prevalence of hearing loss in adolescents based on the data from the 2013 Korea National Health and Nutrition Examination Survey [10]. The results revealed a 22.6% hearing-loss prevalence in adolescents who used earphones in a noisy environment. Those who used the earphones in noise for more than 80 min had a hearing-loss prevalence of 22.3%. Recently, headphones and earphones with a noise-canceling (NC) function were released. As the name infers, the NC function reduces noise. The “canceling” of this noise involves an active noise-control system [11]. How this system works is that a microphone picks up sounds from the environment and inverts them, producing sounds with opposite phase. This way, the sound waves cancel each other out. Examples of earphones and headphones with the NC function include Samsung Galaxy Buds Pro, Apple AirPods Pro, and Bose QuietComfort earbuds and headphones. Previous literature examined the effectiveness and characteristics of the NC function. Liang et al. (2012) examined acoustic characteristics of headphones and earbuds with NC technology and how this technology affected preferred listening levels (PLLs) of 26 individuals [12]. The acoustic characteristics were measured using the Knowles Electronics Manikin for Acoustic Research (KEMAR). For PLLs, participants were asked to adjust the volume while listening to a song with different headphones. The results showed 10–20 dB of noise reduction for frequencies between 1000 and 4000 Hz for warble tones [12]. In the presence of street noise, similar effects were observed for NC headphones but not for the ear buds. Audio devices with the NC function have an advantage in that the function allows individuals to enjoy their listening experiences even more by reducing background noise. However, there is a lack of evidence that suggests the potential of the NC function in hearing protection. The current study explores the effect of the NC function as well as PLLs for two types of earphones: wireless earphones and wired earphones. Additionally, the acoustic characteristics of a stimulus were examined using the KEMAR.

## 2. Materials and Methods

### 2.1. Participants

Individuals aged 19 to 70 years old who did not have any mental and physical disorders were enrolled in the study. Individuals with pure-tone averages (500, 1000, 2000, and 4000 Hz) below 25 dB HL were enrolled in normal hearing (NH) group. Otherwise, individuals were enrolled in the hearing-loss (HL) group. All experimental procedures were approved by Samsung Medical Center’s Institutional Review Board. An informed consent document was obtained from the participants.

### 2.2. Instrumentation

Two types of earphones were utilized for the study: (a) wireless canal type and (b) wired canal type. All of the earphones had the NC function that could be activated or deactivated through a smartphone. “Dynamite” by BTS was chosen as a song to measure PLLs for each participant. It was chosen because it was a popular song; it was on the Billboard Hot 100 chart in 2020. In addition, the song has a small dynamic range, meaning that it is easier for the individuals to adjust the volume. Regarding environmental noises, café and bus noises were used. These noises were downloaded from YouTube, and their volumes were adjusted to 80 dBA using a sound-level meter. The noises were presented through loudspeakers located at 45°, 135°, 225°, and 315° for all tests.

### 2.3. Puretone Audiometry

Puretone audiometry was conducted in a sound booth using an AudioStar Pro (Grason-Stadler, Eden Prairie, MN, USA) audiometer and TDH-39 headphones for all testing frequencies (250–8000 Hz).

### 2.4. Real-Ear Measurement 

For each participant, REMs were performed to examine the sound pressure level (SPL) of the song. REMs are routinely performed as a gold standard in clinical setting for hearing-aid fitting and programming. A probe tube was first marked 30 mm from the open end and inserted into both ear canals. Prior to performing REMs, probe tube calibration was run for all participants. The SPLs were measured in the NC ON and OFF conditions in quiet and noise. The café and bus noises were also presented through the loudspeakers.

### 2.5. Preferred Listening Levels

Each participant’s PLL was also measured using a smartphone (Samsung Galaxy S9). The PLL was defined as a point where the participants felt comfortable listening to the song and did not feel the need to further adjust the volume, which was divided into 15 levels for the Samsung Galaxy S9. Similar to REM, the participants wore the two earphones in a random order with the presentation of the environmental noises as well as the song in a semi-anechoic chamber.

### 2.6. Acoustic Characteristics of the Song Using KEMAR

KEMAR is a manikin used for acoustic research and hearing-device performance testing [13]. The acoustic characteristics of a total of 60 environmental noises (30 bus and 30 café noises) were examined using the two types of the earphones in the NC OFF and ON conditions.

### 2.7. Statistical Analysis

Statistical analysis was performed using IBM SPSS Statistics 26. Comparison of the NC function in the two earphones was completed using the Wilcoxon singed-rank test. The same test was used to compare PLLs before and after the activation of the NC function. For the KEMAR testing, average SPLs in the NC ON and NC OFF for low- (250 and 500 Hz) and whole frequency (200–6000 Hz) were compared to investigate the amount of sound attenuation.

## 3. Results

### 3.1. Demographic Information

A total of 30 individuals participated in the study. Out of the 30 participants, 15 individuals had NH, and 15 individuals had bilateral moderate sensorineural HL. Mean ages of the NH and HL groups were 25.5 (SD = 2.7) years old and 56.2 (SD = 12.6) years old, respectively. Puretone averages of the NH group were 1.4 dB in the right ear and 0.5 dB in the left ear. Puretone averages of the HL group were 40.9 dB in the right ear and 39.1 dB in the left ear.

### 3.2. Noise Attenuation Based on Earphone Type in the NH Group

Table 1 shows the NH group’s mean SPL for the two types of earphones in the presence of bus and café noise. For the bus noise, in the low-frequency range, earphone A had a sound attenuation of 12.0 ± 1.9 dB SPL. Earphone B had sound attenuation of 12.8 ± 2.7 dB SPL. For the whole frequency range, sounds were decreased by 5.4 ± 1.4 dB SPL for earphone A and by 4.0 ± 1.3 dB SPL for earphone B. The Wilcoxon signed-rank test showed statistical significance between the NC OFF and ON conditions for all earphones (*p* < 0.001). For the café noise, earphone A had a sound attenuation of 14.2 ± 2.0 dB SPL in the low-frequency range. Earphone B had a sound attenuation of 12.7 ± 3.2 dB SPL. For the whole frequency range, and earphone A had a sound reduction of 5.1 ± 0.7 dB SPL. For earphone B, sounds were decreased by 4.1 ± 1.0 dB SPL. Statistical significance between the NC OFF and ON conditions were observed for all earphones (*p* < 0.001).

### 3.3. Noise Attenuation Based on Earphone Type in the HL Group

Table 2 For the bus noise, in the low-frequency range, earphone A had a sound attenuation of 11.6 ± 3.6 dB SPL, and earphone B had sound attenuation of 8.5 ± 3.9 dB SPL. For the whole frequency range, sounds were decreased by 5.1 ± 1.6 dB SPL for earphone A and by 2.0 ± 1.7 dB SPL for earphone B. The Wilcoxon signed-rank test showed statistical significance between the NC OFF and ON conditions for all earphones (*p* < 0.001). For the café noise, in the low-frequency range, earphone A had a sound attenuation of 12.7 ± 3.5 dB SPL. Earphone B had sound attenuation of 8.6 ± 4.3 dB SPL. For the whole frequency range, sounds were decreased by 4.3 ± 1.3 dB SPL for earphone A and by 2.8 ± 1.8 dB SPL for earphone B. The Wilcoxon signed-rank test showed statistical significance between the NC OFF and ON conditions for all earphones (*p* < 0.001).

### 3.4. PLLs for NH and HL Groups

Individuals’ preferred listening levels for NC ON and OFF based on earphone types are described in Table 3. In the NH group, for the bus noise, statistical significance was observed between the NC OFF and ON conditions for earphone A and B. For earphone A, the PLL was decreased by 7.0 ± 5.4 levels. For earphone B, the PLL was decreased by 9.0 ± 6.5 levels. When the café noise was presented, sounds were attenuated by 6.0 ± 4.0 levels for earphone A and by 11.0 ± 6.6 levels for earphone B. In the HL group, with the bus noise, individuals’ PLLs were decreased by 7.0 ± 5.2 levels and 12.0 ± 5.3 levels for earphones A and B, respectively. In the presence of café noise, the PLLs were decreased by 4.0 ± 4.1 levels and 9.0 ± 3.9 levels for earphones A and B, respectively. For both groups, statistical significance was observed only for all earphones.

### 3.5. Acoustic Analysis Using KEMAR

**Figure 1:**
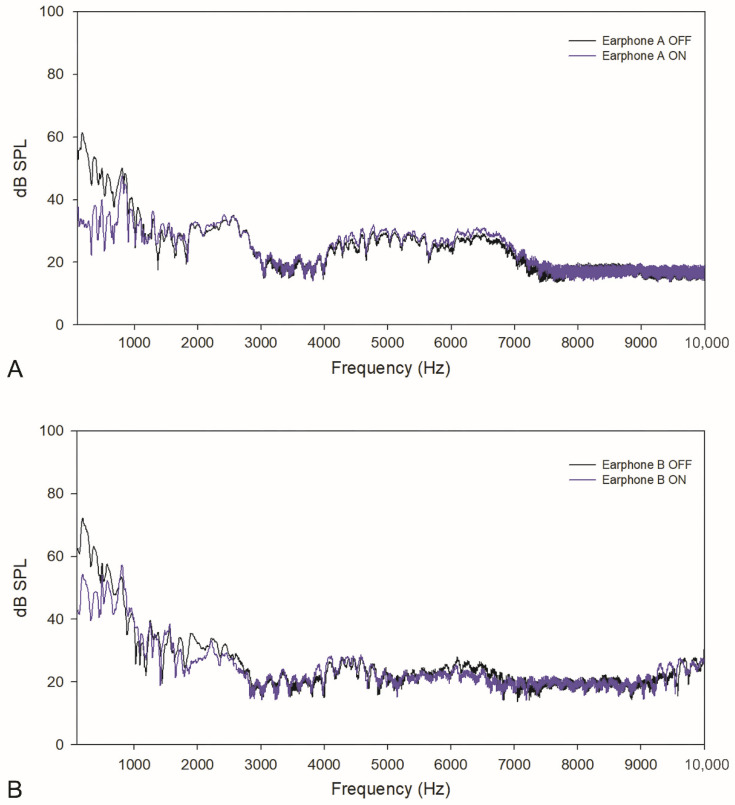
Acoustic characteristics of bus noise in earphone (**A**,**B**).

For the analysis, an average of the 30 signals was used. The KEMAR testing results for bus noise are shown in Figure 1. In the low-frequency range, earphone A had sound attenuation of 18.6 ± 6.1 dB SPL (52.6 ± 4.6 dB SPL with NC OFF and 34.0 ± 2.5 dB SPL with NC ON). Earphone B had sound attenuation of 13.5 ± 8.9 dB SPL (62.5 ± 5.9 dB SPL with NC OFF and 49.0 ± 5.5 dB SPL with NC ON). In the whole frequency range, earphone A had sound attenuation of 8.3 ± 10.4 dB SPL (38.7 ± 14.0 dB SPL with NC OFF and 30.4 ± 5.5 dB SPL with NC ON). Earphone B had sound attenuation of 7.1 ± 8.1 dB SPL (44.4 ± 17.8 dB SPL with NC OFF and 37.3 ± 12.3 dB SPL with NC ON). Overall, both earphone A and B showed reduction in SPLs with the NC function activated. 

**Figure 2:**
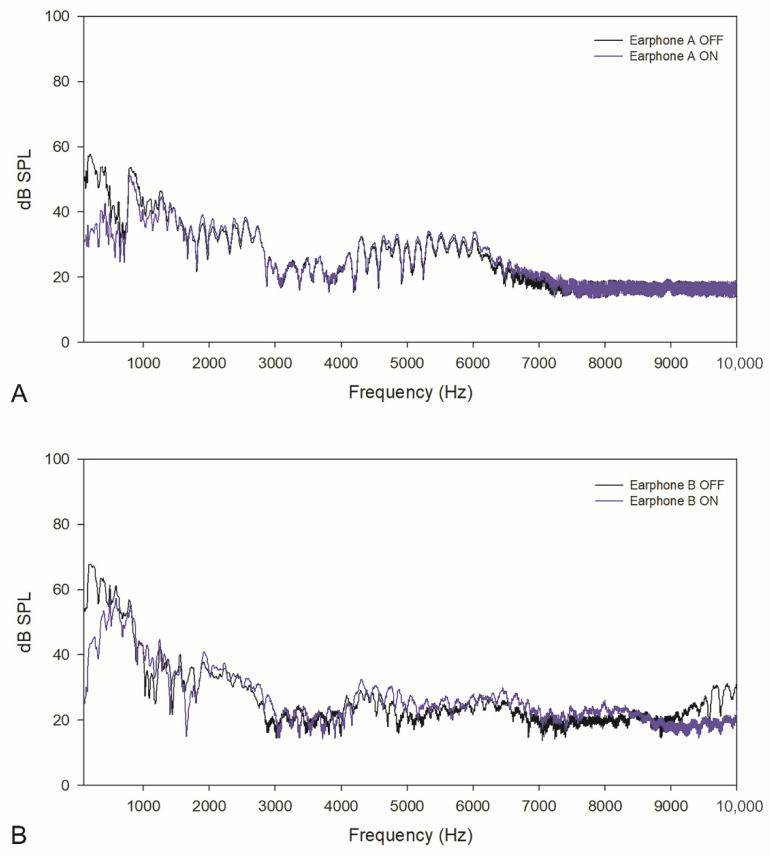
Acoustic characteristics of café noise in earphones (**A**,**B**).

For café noise, as shown in Figure 2, in the low-frequency range, earphone A had sound attenuation of 18.6 ± 6.1 dB SPL (52.6 ± 4.6 dB SPL with NC OFF and 34.0 ± 2.5 dB SPL with NC ON). Earphone B had sound attenuation of 13.5 ± 8.9 dB SPL (62.5 ± 5.9 dB SPL with NC OFF and 49.0 ± 5.5 dB SPL with NC ON). In the whole frequency range, earphone A had sound attenuation of 8.3 ± 10.4 dB SPL (38.7 ± 14.0 dB SPL with NC OFF and 30.4 ± 5.5 dB SPL with NC ON). Earphone B had sound attenuation of 7.1 ± 8.1 dB SPL (44.4 ± 17.8 dB SPL with NC OFF and 37.3 ± 12.3 dB SPL with NC ON). Again, both earphone A and B showed a reduction in SPLs with the NC function activated.

## 4. Discussion

The current study explores how the NC function works in different types of earphones as well as how it impacts PLLs in the presence of café and bus noises. Findings of the study showed that when the NC function is activated in café and bus noises, SPLs of the sound signals were significantly reduced for all types of earphones in the low-frequency range. Similar results were obtained for PLLs: for earphone A and B, which were wireless and wired canal type earphones, PLLs were also significantly decreased with the NC function on. The authors believe that these results were obtained because earphone A and B had rubber tips that could seal the opening of the ear canal. This is consistent with a previous study in 2011 that reported that the structure of the earphone could influence the PLLs and the amount of sound signals coming into the ear canal [14]. In this study, the authors performed REMs utilizing three types of earphones (earbud, ear canal, and on-the-ear earphone) to measure PLLs in quiet and noise for 25 adults. In quiet, the ear canal earphone had the lowest PLLs (60.3 ± 12.9 dB SPL). In noise, the on-the-ear earphone had the largest PLLs (77.5 ± 11.2 dB SPL). The authors mentioned that sealing the ear canal would prevent noise coming into the ear canal. This leads to better signal-to-noise ratio, and therefore, listeners could easily enjoy music at a lower volume [14]. Another factor that could have influenced the PLLs was loud noise [4,9,15]. Williams (2005) reported that a high volume of environmental noises used in the study led to the loudest volume settings for the participants [4]. Hodgetts et al. (2007) also mentioned that PLLs were higher for noise conditions than those for the quiet condition [9]. When it comes to noise exposure and hearing loss, noise levels and duration of exposure are important [16,17]. The U.S. Environmental Protection Agency and the World Health Organization reported that one is at the risk of noise-induced hearing loss if he or she is exposure to sounds at 85 dBA for eight hours. With the 3 dB rule, this safe exposure time becomes half for every 3 dB the sound intensity increases. For example, at the sound level of 88 dBA, one’s safe listening time is four hours, and at 115 dB, which is the sound level equivalent to leaf blower and rock concert, the safe listening time is only about 30 s. Thus, it is essential to examine if the NC function can help individuals protect their hearing and how. Considering the fact that the volume levels decreased by six to twelve levels for bus noise and by four to eleven levels for café noise with the activation of the NC function, the findings of this study suggest that the NC function could possibly contribute to hearing protection. Subsequent studies with a higher number of participants and inclusion of other tests, such as otoacoustic emission tests, are necessary to assess the potential of the NC earphones for hearing protection. It would be helpful to investigate hearing characteristics of individuals who use earphones or headphones with and without the NC technology. Matching the age groups would lead to audiometric equality. Lastly, as there are different types of NC systems (i.e., passive NC, active NC, and adaptive active NC) [18], it would also be beneficial to explore the amount of sound attenuation for each system and its feasibility to be used as a tool for hearing protection.

## Figures and Tables

**Table 1:** NH group’s mean and standard deviation of SPLs for the two types of earphones in bus and café noises.

Frequency Range	Noise	Earphone Type	SPL Mean (SD)		*p*-Value
			NC OFF	NC ON	
Low	Bus	A	66.3 ± 1.4 dB SPL	54.2 ± 2.3 dB SPL	<0.001
		B	66.6 ± 2.4 dB SPL	53.8 ± 4.2 dB SPL	<0.001
	Café	A	61.2 ± 1.6 dB SPL	47.0 ± 2.9 dB SPL	<0.001
		B	61.8 ± 1.8 dB SPL	49.8 ± 3.4 dB SPL	<0.001
Whole	Bus	A	47.2 ± 2.1 dB SPL	41.8 ± 2.5 dB SPL	<0.001
		B	47.5 ± 1.8 dB SPL	43.6 ± 2.5 dB SPL	<0.001
	Café	A	45.8 ± 1.8 dB SPL	40.7 ± 2.0 dB SPL	<0.001
		B	46.5 ± 1.9 dB SPL	42.4 ± 2.1 dB SPL	<0.001

**Table 2:** HL group’s mean and standard deviation of SPLs for the two types of earphones in bus and café noises.

Frequency Range	Noise	Earphone Type	SPL Mean (SD)		*p*-Value
			NC OFF	NC ON	
Low	Bus	A	69.0 ± 2.2 dB SPL	56.7 ± 5.6 dB SPL	<0.001
		B	69.8 ± 1.3 dB SPL	61.3 ± 4.9 dB SPL	<0.001
	Café	A	63.0 ± 3.3 dB SPL	50.3 ± 6.2 dB SPL	<0.001
		B	64.6 ± 1.0 dB SPL	56.0 ± 4.9 dB SPL	<0.001
Whole	Bus	A	51.5 ± 2.6 dB SPL	45.7 ± 4.0 dB SPL	<0.001
		B	52.7 ± 4.0 dB SPL	50.7 ± 5.1 dB SPL	<0.001
	Café	A	48.6 ± 3.2 dB SPL	44.2 ± 3.9 dB SPL	<0.001
		B	52.0 ± 3.5 dB SPL	49.0 ± 5.1 dB SPL	<0.001

**Table 3:** PLLs when the NC function is turned on and off in both groups.

Group	Noise	Earphone Type	PLLs Mean (SD)		*p*-Value
			NC OFF	NC ON	
NH	Bus	A	50.0 ± 11.4 level	43.0 ± 13.3 level	<0.01
		B	39.0 ± 13.7 level	48.0 ± 14.1 level	<0.01
	Café	A	49.0 ± 11.2 level	43.0 ± 10.0 level	<0.01
		B	46.0 ± 16.8 level	35.0 ± 15.5 level	<0.01
HL	Bus	A	48.0 ± 9.9 level	42.0 ± 10.4 level	<0.01
		B	51.0 ± 13.2 level	39.0 ± 12.2 level	<0.001
	Café	A	46.0 ± 12.4 level	42.0 ± 10.4 level	<0.01
		B	46.0 ± 11.6 level	37.0 ± 10.6 level	<0.001

## Data Availability

The data supporting the findings of this study are available from the corresponding author, I.-J.M., upon reasonable request. The data were not publicly available because of ethical considerations.
